# Safety and Biovigilance in Organ Donation (SAFEBOD): Protocol for a Population-Based Cohort Study

**DOI:** 10.2196/18282

**Published:** 2020-10-26

**Authors:** Brenda Rosales, James Hedley, Nicole De La Mata, Claire M Vajdic, Patrick Kelly, Kate Wyburn, Angela C Webster, The SAFEBOD Study Group

**Affiliations:** 1 Sydney School of Public Health The University of Sydney Camperdown Australia; 2 Centre for Big Data Research in Health University of New South Wales Sydney Australia; 3 Central Clinical School Faculty of Medicine and Health The University of Sydney Sydney Australia; 4 Renal Medicine Royal Prince Alfred Hospital Sydney Australia; 5 Renal Medicine and Transplantation Westmead Hospital Westmead Australia; 6 See Acknowledgments

**Keywords:** transplant recipients, organ donor, transplant, neoplasms, infectious, disease transmission, safety, biovigilance, organ, surgery, cohort study

## Abstract

**Background:**

Tension lies between the need to increase access to organ transplantation and the equally urgent need to prevent inadvertent transmission of infectious diseases or cancer from organ donors. Biovigilance, or the evaluation of potential donors, is often time-pressured and may be based on incomplete information.

**Objective:**

The Safety and Biovigilance in Organ Donation (SAFEBOD) study aims to improve estimates of infection and cancer transmission risk and explore how real-time data access could support decision-making.

**Methods:**

We will link existing donor referral, actual donor, recipient, and health-outcome data sets from 2000-2015 in New South Wales. Organ donor data sets will include the Organ Donor Characterizing Risk-Profile of Donors Study, the National Organ Matching System, the Australian and New Zealand Organ Donor Register, and the Australian and New Zealand Living Donor Kidney Register. Recipient data sets will include the Australian and New Zealand Dialysis and Transplant Register, the Australian and New Zealand Cardiothoracic Register, the Australian and New Zealand Islet and Pancreas Register, and the Australian and New Zealand Liver Transplant Register. New South Wales health outcome data sets will include HIV and AIDS Notifications and Surveillance Data, the Notifiable Conditions Information Management System, Admitted Patient Data Collection, Emergency Department Data Collection, the Central Cancer Registry, and the Cause of Death Data Collection. We will link organ donors to transplant recipients and health outcomes data sets using probabilistic data-matching based on personal identifiers. Transmission and nontransmission events will be determined by comparing previous cases in donors and posttransplant cases in recipients. We will compare the perceived-risk at referral with the verified risk from linked health outcome data sets and the odds of cancer or contracting an infectious disease in organ recipients from donors based on their transmission-risk profile and estimate recipient survival by donor transmission risk group.

**Results:**

Data were requested from each of the listed registries in September 2018, and data collection is ongoing. Linked data from all listed data sets are expected to be complete in September 2020.

**Conclusions:**

The SAFEBOD study will overcome current limitations in organ donation by accessing comprehensive information on referred organ donors and recipients in existing data sets. The study will provide robust estimates of disease transmission and nontransmission events based on recent data. It will also describe the agreement between perceived risk estimated at the time of referral and verified risk when all health outcome data are accessible. The improved understanding of transmission and nontransmission events will inform clinical decisions and highlight where current policies can be revised to broaden the acceptance of deceased donors.

**International Registered Report Identifier (IRRID):**

DERR1-10.2196/18282

## Introduction

**Figure 1:**
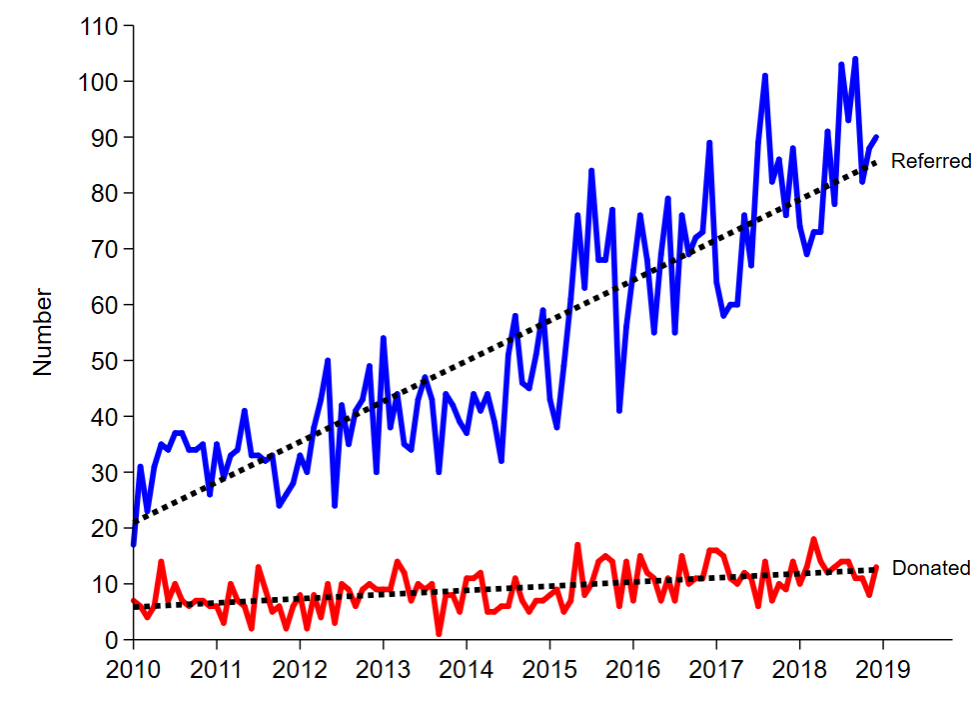
Number of organ donor referrals and actual donations in New South Wales, 2010-2018 [3].

**Figure 2:**
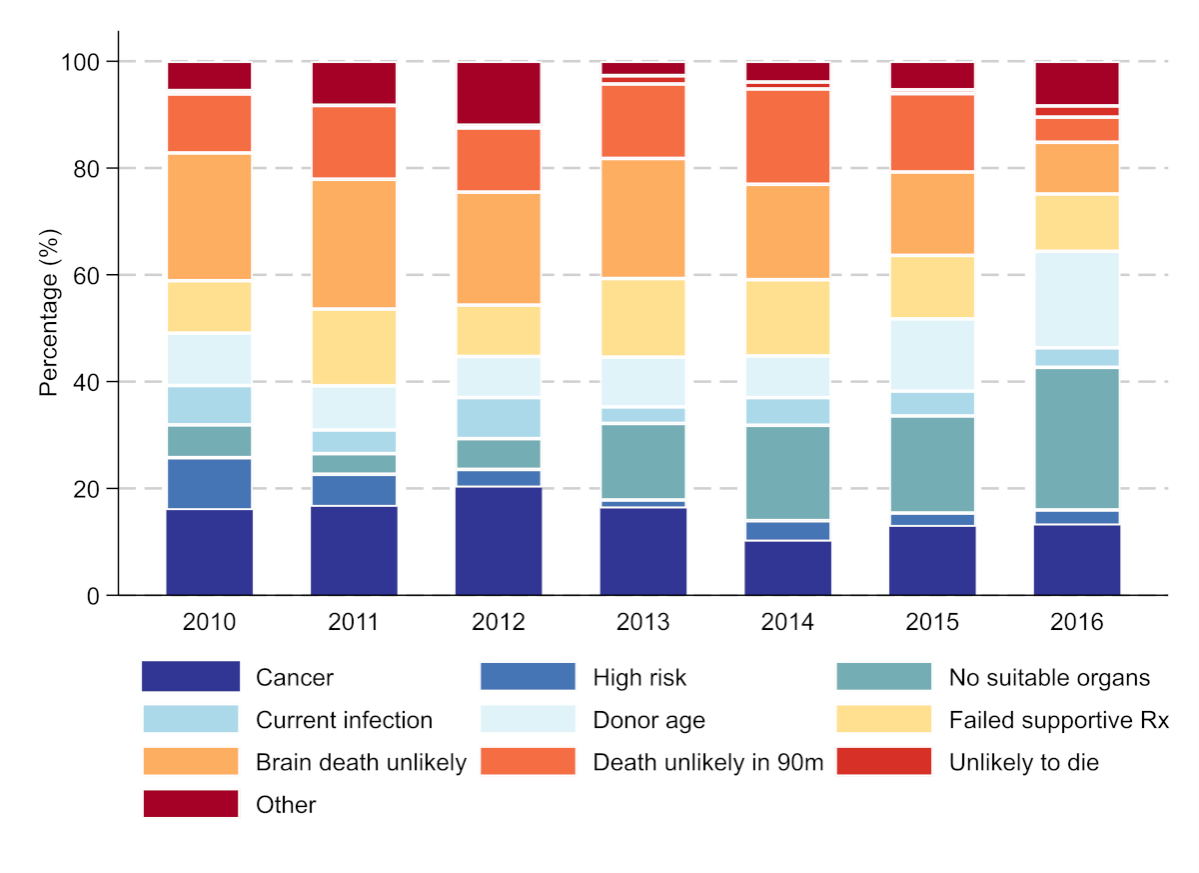
Primary reason donor referrals were found not medically suitable. Reasons of possible biovigilance concern highlighted in blue.

Biovigilance is intended to avoid inadvertent transmission of infectious disease or cancer from organ donors and is a central concern in transplant programs globally. In conflict with these safety concerns is the excess morbidity and mortality experienced by people with end-organ disease on transplant waiting lists. In Australia, increasing the organ donation rate is a national priority. The Australian Government formed the Organ and Tissue Authority (OTA) in 2009. The OTA’s purpose is to increase the capability and capacity within the health system to maximize donation rates and to raise community awareness and stakeholder engagement across Australia to promote organ and tissue donation. Since 2009 the number of deceased organ donors has more than doubled, and the number of transplant recipients has increased by 75% [1]. However, the number of people in need of an organ transplant outweighs the number of organs available. At the end of 2017, 1388 patients with end-stage chronic disease remained active on the transplant waiting list (964 kidney, 171 liver, 80 heart, 108 lung, 65 pancreas, 3 intestine) [2]. The number of referrals of potential donors has increased exponentially over time, but the number of referrals who go on to become donors has increased modestly in comparison [3,4]. Thus, the proportion of total donor referrals who proceed to donation has decreased over time (Figure 1 [3]). A large proportion of donor referrals do not proceed to donation due to biovigilance concerns (Figure 2). Therefore, initiatives to increase the number of referred potential donors whose organs can be safely donated and transplanted are vital.

Decisions about donor medical suitability and biovigilance are informed by sparse data and tend to be risk averse. Using published medical literature to understand transmission risk is problematic due to publication bias (resulting in more events of disease transmission being reported than events where there was no disease transmission). Published transmission events are likely to be a biased subset of the global transplantation experience. Current estimates of transmission risk for bloodborne viruses or malignancies in organ donation include confirmed transmission events [5,6]. However, the proportion of organs transplanted from donors with a perceived increased risk of infection or malignancy, but there was no transmission, is unknown. The need for an evidence base to guide decision-making led to the formation of the NOTIFY project as a joint venture between the World Health Organization (WHO) and the Italian National Transplant Centre. NOTIFY collates a biovigilance database and recommends biovigilance systems in organ donation and transplant report all serious adverse events nationally [7].

Risk stratification recommendations for different infectious diseases and cancers are often complex, may not be derived for use in a transplantation setting, and may not be readily accessible in real-time to clinicians making decisions about donor safety. Inconsistencies in decisions about the medical suitability of referred donors suggest considerable clinical uncertainty [3]. For example, complex guidelines and uncertainty around the risk of transmission of primary brain malignancies from donor to recipient may have resulted in 23 missed donor opportunities where the transmission risk was subsequently ascertained to be low [4]. The inclusion of these donors would have increased the donor pool by 3.1%.

The Safety and Biovigilance in Organ Donation (SAFEBOD) study aims to estimate infection and cancer transmission risk and provide insight into how real-time access to linked existing data could support decision-making.

## Methods

SAFEBOD is a cohort study using data linkage of existing state and national administrative health data sets. These data sets will establish estimates of the biovigilance risk of living and deceased organ donors, and potential deceased donor referrals that do not proceed, in New South Wales (NSW).

### Aims

The study’s primary objective is to develop clearer estimates of disease transmission in organ donation and transplant. Specifically, the study aims to (1) identify organ donors and recipients with recorded cancer or infectious disease, (2) determine the agreement between medical history ascertained at the time of donor referral (perceived risk) and that collated from existing mandated health data sets (verified risk), (3) identify suspected cases of donor-recipient disease transmission and nontransmission based on presence or absence of the corresponding disease in donor and recipient verified records. The study’s findings will be used to develop decision support recommendations and resources for clinicians making donation decisions in NSW and beyond.

### Public Health Register

The database generated in this project will be established as the Biovigilance in Organ Donation and Transplantation Register (Biovigilance Register) under state law by the NSW Ministry of Health. The Public Health Act 2010 permits the linkage of existing health data sets to facilitate the identification and monitoring of risk factors for diseases or conditions that have a substantial adverse impact on the population and, to facilitate the care, treatment, and the follow up of persons who have diseases or have been exposed to diseases of public safety [8]. The sponsor of the Biovigilance Register is the NSW Chief Health Officer and Deputy Director-General, Population and Public Health. The data custodian of the Biovigilance Register is the Associate Director, Epidemiology and Biostatistics, Centre for Epidemiology and Evidence.

### Population

The study will consist of four groups of participants: (1) Donor referrals that did not proceed to donation, (2) Deceased donors, (3) Living donors, and (4) Recipients of any organs from 2 and 3, for all solid organs used or procured for transplantation in NSW, 2000-2015.

### Data Sources

**Figure 3:**
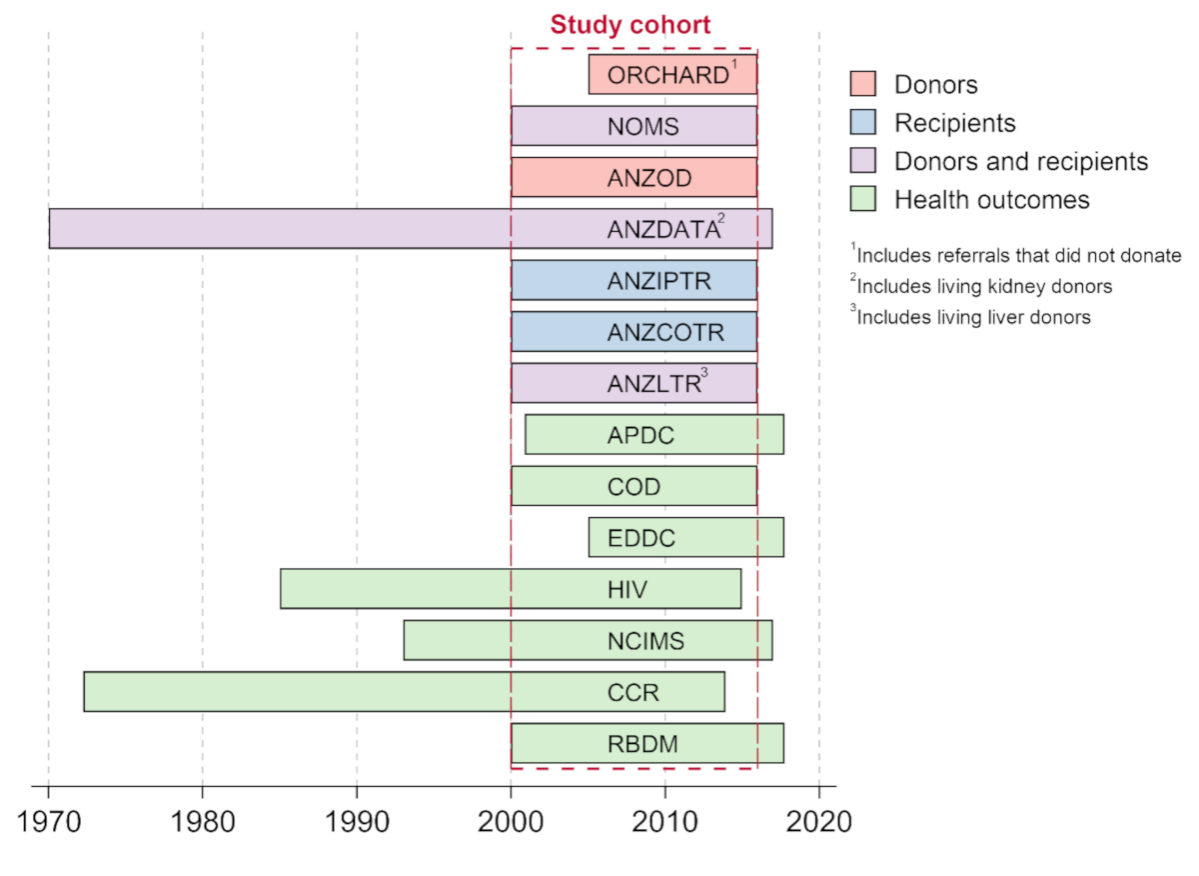
Data sources included in the SAFEBOD study with date ranges of the data requested. Personal identifiers will be requested for individuals referred for donation, donated, or transplanted in New South Wales, 2000-2015 (dotted box) to define the study cohort.

The Biovigilance Register will source information from organ donor referral, living and deceased donor, recipient registries, and health outcome data sets (Figure 3). A description of each data set and the date range of available data follows.

#### Organ Donor Referral, Deceased Donor, and Living Donors

##### Organ Referral Characterisation Database (ORCHARD), 2005-Present

Developed by nephrologists Angela Webster and Kate Wyburn in association with the NSW Organ and Tissue Donation Service (OTDS) using the OTDS registry of referral logs for organ donation in NSW, ORCHARD records all donor referrals in NSW, regardless of referral outcome. The database includes information about perceived cases of cancer and infectious diseases in referrals who do and do not proceed to donation.

##### The National Organ Matching System (NOMS) Database, 2000-Present

The allocation of organs from a deceased donor to patients on the waiting list is determined by ranking generated by a computer program administered by the Australian Red Cross. NOMS holds additional identifiers for NSW donors and recipients useful for data linkage and immune profiles of donor and recipient pairs used to calculate their matching scores.

##### Australia and New Zealand Organ Donor (ANZOD) Registry, 1989-Present

This registry collects and records data on all organ donors within Australia and New Zealand. The database is essential to linking donor and recipient pairs identified in the donor and recipient registries.

##### Australia and New Zealand Living Kidney Donor Register, 2004-Present

This registry collects and records data on all living kidney donors within Australia and New Zealand.

#### Organ Transplant Recipients

##### Australia and New Zealand Dialysis and Transplant (ANZDATA) Registry, 1977-Present

Records information for all people in Australia and New Zealand receiving treatment for end-stage renal failure, including those who have received a kidney transplant, updated annually.

##### Australian and New Zealand Islet and Pancreas Transplant Recipient Registry (ANZIPTR), 1984-present

Records all islet and pancreas transplants performed in Australia and New Zealand.

##### Australia and New Zealand Cardiothoracic Transplant Registry (ANZCOTR), 1984-Present

This registry contains every heart, heart/lung, and lung transplant performed in all six Australia and New Zealand Cardiothoracic Transplant centers.

##### Australia and New Zealand Liver Transplant Registry (ANZLTR), 1985-Present

This collaborative effort of the liver transplant units in Australia and New Zealand collects data on all patients listed for liver transplantation and their subsequent outcomes.

#### Health Outcomes

##### NSW Admitted Patient Data Collection (APDC) (Public Hospitals), and the NSW Inpatient Statistics Collection (Private Hospitals), 2001-2018

These comprise a census of all admitted patient services provided by NSW public hospitals, public psychiatric hospitals, public multi-purpose services, private hospitals, and private day procedure centers. It covers demographic information and information on diagnoses, procedures, and hospital care for every hospital separation in NSW. Admitted patient data are collected under administrative arrangements with public hospitals and the Private Health Facilities Act 2007 for private hospitals.

##### NSW Cause of Death Unit Record File (COD), 1985-2016

The Australian Coordinating Registry provides the COD on behalf of the data custodians, the Registry of Births, Deaths, and Marriages, and the State Coroner. The COD data set includes death registration information pertaining to all deaths occurring in NSW and includes demographic information, cause of death, and place of death as recorded either through the death registration process or by coroners. This information has been supplemented with codes derived by the Australian Bureau of Statistics, including the International Classification of Disease Codes.

##### NSW Emergency Department Data Collection (EDDC), 2005-2018

The EDDC registry records demographic and emergency treatment-related information for every person who presents to participating public emergency departments in NSW, including all emergency departments in metropolitan public hospitals and rural base hospitals. Information on emergency department attendances is collected under administrative arrangements with public hospitals.

##### HIV and AIDS Notifications and Surveillance Data Set (HIV), 1985-2014

HIV is notifiable to the Ministry of Health under the NSW Public Health Act 2010. Notifications of HIV are received from pathology laboratories and compiled in the HIV Notifications and Surveillance Dataset.

##### NSW Notifiable Conditions Information Management System (NCIMS), 1993-2017

The NCIMS manages the surveillance and reporting of diseases and conditions notifiable under the NSW Public Health Act 2010. The NSW Ministry of Health receives notifications of communicable diseases from general practitioners, hospitals, and pathology laboratories. All notifiable conditions included in NCIMS will be included in the Biovigilance Register except for adverse events following immunization and lead poisoning.

##### NSW Central Cancer Register (CCR), 1972-2015

The CCR records all new diagnoses of invasive cancer and in-situ breast cancer and melanoma in NSW residents but does not capture cancer recurrences.

##### NSW Registry of Births, Deaths, and Marriages (RBDM) Death Registrations, 1985-2018

This registry includes all deaths occurring in NSW. Demographic information and particulars of each death, including the cause of death, are recorded.

##### NSW Tuberculosis Contact Treatment Chest Clinics (TB), 2000-2015

Chest Clinic databases across NSW hold records of tuberculosis cases and people treated as contacts of cases.

##### South Eastern Area Laboratory Services (SEALS), 2008-Present

SEALS performs serological and nucleic acid testing for bloodborne viruses in organ donation in NSW. It holds records for HBV, HCV, and HIV results for intended and actual donors in NSW. These records are sought to complement and verify the HBV and HCV findings collated from other outcomes, donor, and recipient registries.

### Data Linkage

**Figure 4:**
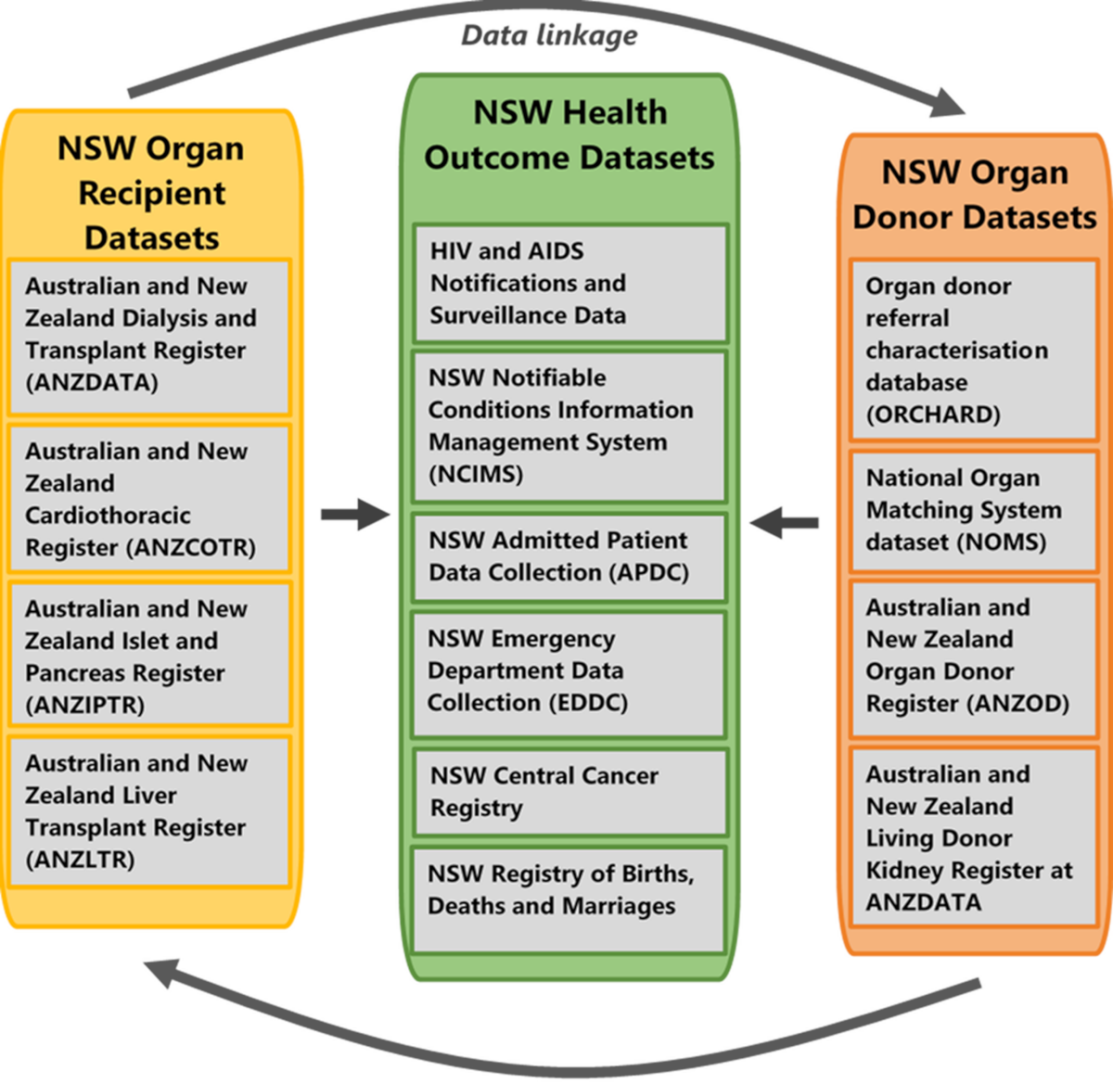
Data sources and data linkage process between organ recipient data sets, organ donor data sets, and health outcome data sets in New South Wales.

Data linkage will be performed by the NSW Ministry of Health dedicated data linkage service, the Centre for Health Records Linkage (CHeReL). The CHeReL uses a separation model for data integration in order to maintain patient confidentiality [9]. Personal identifiers are split from health information for each data set. Data custodians will send demographic data, including name, sex, date of birth, and address, if available to the CHeReL. These identifiers will be used to link individuals across data sets. The CHeReL will then randomly assign a unique identifier to individuals across data sets and match donor and recipient records. Linkage will be probabilistic for all data sets except ANZDATA, ANZOD, and the Living Kidney Donor registers, which are already deterministically linked [10]. The CHeReL will return unique individual identifiers to the data custodians, who then send the health-related data with the unique identifier back to the NSW Ministry of Health, who will create the Biovigilance Register (Figure 4). Data shared with the research team at The University of Sydney will be de-identified to maintain patient confidentiality. The risk of reidentification of patients is very low with the information held in the Biovigilance Register.

### Outcomes

#### Agreement of Perceived and Verified Risk

The possible outcomes of decisions made according to perceived risk compared to verified risk are outlined in Table 1. When perceived risk and verified risk are concordant, donors are used effectively and efficiently. Donors perceived as high risk who truly pose a biovigilance risk are declined (Outcome 1), or their organs may be used with recipient consent, appropriate prophylaxis, and surveillance. In cases of known transmission (Outcome 2), the risk of transmission and recipient outcome is mitigated by early recognition and treatment, and the (infectious) disease may not manifest, a known nontransmission (Outcome 3). The most likely clinical scenario for donors is a perceived low risk, which can be verified as a known absence of biovigilance risk (Outcome 8).

When donors are incorrectly classified, they are not used effectively or efficiently. Incorrect classification occurs when verified low-risk donors are declined because of a high perceived risk (Outcome 6), or recipients are not perceived as at-risk of unknown or nontransmission (Outcomes 4 and 5) or exposed to unnecessary treatments and tests when donors are believed to be a risk for transmission when they are low risk (Outcome 7).

**Table 1:** Summary of possible donation outcomes and transmission events, according to perception and verification of donor biovigilance risk. Top left and bottom right: perceived risk and verified risk are concordant, and the donor is correctly classified. Top right and bottom left: perceived risk and verified risk are discordant, and the donor is incorrectly classified.

Perceived donor risk	Verified donor risk	
	High	Low
High	Donor declined Known transmission Known nontransmission	Donor declined Misclassified known nontransmission
Low	Unknown transmission Unknown nontransmission	Known absence of biovigilance risk

#### Suspected Cases of Donor-Derived Disease Transmission

Two statisticians will review donor and recipient records to identify suspected cases of donor-derived transmission events for infectious diseases and malignancies. Suspected cases will include recipients first diagnosed with the condition post transplant where (1) the donor was known to have the condition at the time of donation or (2) the donor was not known to have the condition at the time of donation, and more than one recipient from the same donor was diagnosed with the same condition. A standardized algorithm will be used to classify the likelihood of the transmission event being donor-derived from excluded to possible/probable/proven, as used by the Organ Procurement Transplant Network Disease Transmission Advisory Committee [11,12]. This algorithm considers several criteria for classification, including laboratory evidence in the donor, all recipients of the same donor, and pretransplant laboratory evidence of negative findings in the recipient before transplant and use of active prophylaxis or treatment. For infectious diseases, we will also consider the time from transplant to diagnosis to distinguish between donor-derived transmission events and de novo infections.

### Statistical Analysis

Estimates of transmission through organ transplantation will be based on the recorded cases using linked health data in donors and recipients. Information from the linked health data will be used to identify known and unknown cases of donor infectious diseases and/or malignancies and linked to respective recipients to determine disease transmission and nontransmission events in patients transplanted in NSW.

Donor “perceived” risk for cancers and infectious diseases will be compared to “verified” risk using proportions (95%CI) and McNemar tests, and agreement will be assessed using the Kappa statistic. Cox or logistic regression models will be fitted to compare the hazard/odds of cancer or contracting an infectious disease in organ recipients from donors classified by the four transmission risk groups (classified according to Table 1). Other risk factors of recipient cancer or infection will be adjusted for in the analyses. Recipient survival by donor transmission risk groups will be summarised using Kaplan–Meier survival curves, and hazard ratios (95% CI) estimated using Cox regression models. Additional average life-years gained by using organs from donors stratified by risk of cancer transmission will be estimated from the area under the survival function curve up to 10 years after transplantation. All analyses will be conducted using STATA, R, or SAS statistical software programs.

## Results

The SAFEBOD study was funded in 2016 by the Office of the Chair, NSW Ministry of Health. We received approval from the University of Sydney Human Research Ethics Committee (HREC 2016/758) on September 13, 2016. Data were requested from the listed registries in September 2018 and is ongoing. Linked data from all listed data sets is expected in September 2020.

### Declarations

The SAFEBOD study was approved by the University of Sydney Human Research Ethics Committee (HREC 2016/758) on September 13, 2016. It includes the approval of data collection for three populations: deceased organ donors, organ transplant recipients, and living organ donors. HREC approved a waiver of consent to participate for deceased organ donors. HREC approved disclosure of health-related information for organ transplant recipients and living organ donors under the Public Health Act (1998) and the management of health services activity (HPP 10 (1) or 11(1)). The linked donor, recipient, and health-related data sets will form the Bioviglance in Organ Donation and Transplantation Register under state law by the NSW Ministry of Health, established by the authority of the Chief Health Officer for epidemiological data under the Public Health Act 2010. The Register will be housed by the NSW Ministry of Health and provided to the research group for investigation. The researchers will follow the NSW Health policy directive on Data Collections—Disclosure of Unit Record Data for Research or Management of Health Services (PD2015_037, September 15, 2015).

### Availability of Data and Material

The data that support the findings of this study are available from the NSW Ministry of Health, Office of the Chair, but restrictions apply to the availability of these data, which were used under license for the current study, and so are not publicly available. Aggregate data are, however, available from the authors after publication of findings, upon reasonable request and with permission of the NSW Ministry of Health, Office of the Chair, and all other data custodians for the data sets named above.

## Discussion

Organ donation remains a scarce resource despite a dramatic increase in organ donor referrals in the past ten years. Many donor referrals are declined due to the perceived risk of transmission of infectious disease or malignancy at the time of referral. Often, data collected at the time of referral is incomplete due to a lack of access to medical records to ascertain a donor’s medical history within donation time-frame constraints and limits the opportunity for estimating biovigilance outcomes. Current estimates of disease transmission in organ donation and transplantation are biased towards transmission events and do not reliably capture nontransmission, leading to the over-estimation of biovigilance risk posed by referred donors.

Our proposed study will overcome these limitations using data linkage to develop clearer estimates of donor disease transmission and nontransmission in NSW. Our study cohort is uniquely placed for this work due to high-quality state data and linkage infrastructure and the use of the unique data set of contemporaneous potential donors, which are not recorded by deceased donor registries. Furthermore, it will describe the agreement between perceived risk of referred donors based on data collected at the time of referral and verified risk when mandatory health outcomes data are available. These results may reveal additional information available in health outcome data sets and support the use of real-time linkage at the time of donor referral to ascertain transmission risk.

Some practical and operational issues involved in performing the study arise from the use of state data in a nationally shared deceased donor organ allocation program. Cases will arise where deceased donor organs from interstate or NSW donor organs have been allocated to interstate recipients. In these cases, we will be unable to verify the perceived risk at the time of donation with outcomes collated from linked health records. However, these cases can contribute to the estimations of disease transmission based on donor histories collected at the time of referral and recipient outcomes recorded by transplant registries. Additionally, we will be unable to verify donor risk or censor those lost to follow-up (except kidney recipients in ANZDATA), living donors, and recipients who move interstate or overseas.

Findings from the SAFEBOD study will highlight where the current policy can be revised to accept more donors and increase transplant rates. Clear estimates of transmission risk will assist in clinical decision-making at the time of donor referral and may also be useful in conversations with potential recipients.

## Abbreviations

ANZCOTR: Australia and New Zealand Cardiothoracic Transplant Registry
ANZDATA: Australia and New Zealand Dialysis and Transplant Registry
ANZIPTR: Australia and New Zealand Islet and Pancreas Transplant Recipient Registry
ANZLTR: Australia and New Zealand Liver Transplant Registry
ANZOD: Australia and New Zealand Organ Donor Registry
APDC: NSW Admitted Patient Data Collection
CCR: NSW Central Cancer Register
CHeReL: Centre for Health Record Linkage
CI: chief investigator
COD URF: NSW Cause of Death Unit Record File
EDDC: NSW Emergency Department Data Collection
HIV: HIV Notifications and Surveillance Dataset
NCIMS: NSW Notifiable Conditions Information Management System
NOMS: National Organ Matching System
NSW: New South Wales, Australia
ORCHARD: Organ Donor Referral Characterisation Database
OTDS: Organ and Tissue Donation Service
PDDT: potential donor-derived disease transmission
RBDM: NSW Registry of Births, Deaths, and Marriages
SAFEBOD: Safety and Biovigilance in Organ Donation Study
SEALS: South Eastern Area Laboratory Services
TB: NSW Tuberculosis Contact Treatment Chest Clinics
WHO: World Health Organization
